# Molecular Pathways Affected by Sulfonylpurine Derivatives in 2D and 3D HeLa Cell Models

**DOI:** 10.3390/molecules30234659

**Published:** 2025-12-04

**Authors:** Marijana Leventić, Josipa Matić, Dijana Pavlović Saftić, Željka Ban, Biserka Žinić, Ljubica Glavaš-Obrovac

**Affiliations:** 1 Department of Medicinal Chemistry, Biochemistry and Laboratory Medicine, Faculty of Medicine Osijek, Josip Juraj Strossmayer University of Osijek, Huttlerova 4, 31000 Osijek, Croatia; 2 Laboratory for Biomolecular Interactions and Spectroscopy, Division of Organic Chemistry and Biochemistry, Ruđer Boškovicć Institute, Bijenička Cesta 54, 10000 Zagreb, Croatia; jmatic@irb.hr (J.M.); dsaftic@irb.hr (D.P.S.); zeljka.ban@irb.hr (Ž.B.); bzinic@irb.hr (B.Ž.)

**Keywords:** 3D Hela model, sulfonylpurine derivatives, Akt signaling pathway, miRNAs

## Abstract

This study investigates two sulfonylpurine derivatives, Pur-6-NH_2_-SS and Pur-6-Mor-SS, which contain amino and morpholino substituents, for their anticancer potential in 2D and 3D models of human cervical adenocarcinoma (HeLa) cells. Cell cycle distribution, apoptosis, mitochondrial membrane potential, and ROS accumulation were evaluated by flow cytometry. Both Pur-6-NH_2_-SS and Pur-6-Mor-SS reduced the proportion of cells in the G0/G1 phase (to 39.65 ± 5.59% and 28.25 ± 1.20%, respectively) when compared with untreated cells. Pur-6-NH_2_-SS additionally increased the proportion of cells in the S phase (7.41 ± 0.32%), whereas Pur-6-Mor-SS increased the number of cells in subG0 (21.05 ± 6.15%). Additionally, Pur-6-NH_2_-SS triggered early apoptosis in 79.6 ± 8.5% of cells, accompanied by mitochondrial membrane depolarisation in 64.3 ± 9.0%. In comparison, Pur-6-Mor-SS elicited an even stronger apoptotic response, inducing early apoptosis in 87.4 ± 15.6% of cells and mitochondrial membrane potential disruption in 86.8 ± 9.0%, relative to untreated cells. RT-PCR analysis assessed the expression of key regulators, including miR-21, miR-210, and genes involved in survival and stress-response pathways (*Akt*, *CAIX*, *caspase-3*, and *cytochrome C*). In the 2D model, both derivatives increased *CAIX*, *Akt*, and *Cyp C* expression compared with untreated cells. In contrast, *p53* expression remained unchanged in Pur-6-NH_2_-SS-treated cells and was slightly decreased in Pur-6-Mor-SS-treated cells. Casp3 expression was slightly elevated following Pur-6-NH_2_-SS treatment and remained nearly unchanged in Pur-6-Mor-SS-treated cells. In the 3D model, Pur-6-NH_2_-SS exerted a stronger inhibitory effect on *CAIX*, *Akt*, *p53*, *Cyp C*, and *Casp3* expression than Pur-6-Mor-SS, which showed weaker inhibition overall. Both derivatives had a comparable impact on miR-21 and miR-210 expression in 2D and 3D HeLa models. These findings provide mechanistic insight into amino- and morpholino-substituted sulfonylpurine derivatives and highlight how 2D and 3D tumour models influence drug response, offering a basis for further development of purine-based anticancer agents.

## 1. Introduction

Purine derivatives, as structural mimics of endogenous biomolecules, represent a versatile and well-established scaffold for anticancer drug design [1,2]. Several purine analogues have been approved by the Food and Drug Administration for cancer treatment, including mercaptopurine, thioguanine, fludarabine phosphate, cladribine, pentostatin, clofarabine, and nelarabine [3,4,5]. Synthetic sulfonylpurine derivatives, especially those modified with amino and morpholino substituents, have emerged as potential candidates for antitumor therapy because of their ability to interfere with signalling pathways that regulate cell survival, apoptosis, and stress response [6,7]. However, despite promising preliminary results, the exact mechanisms of action of these derivatives, particularly in the context of different tumour microenvironments, remain to be elucidated.

The dimensionality of cell cultures influences cellular responses, making 3D spheroids particularly useful for studying tumour-like behaviour not observable in 2D systems [8,9,10,11]. While 2D cultures are standard, 3D models more accurately reproduce key features of the tumour microenvironment. Spheroid 3D cultures recreate tumour-like gradients, providing a more realistic model for testing anticancer agents [12].Under hypoxic conditions, cancer cells activate survival pathways such as phosphatidylinositol-3-kinase (PI3K)/Akt, mammalian target of rapamycin (mTOR), mitogen-activated protein kinases (MAPK), and nuclear factor kappa-light-chain-enhancer of activated B cells (NF-κB) signaling cascades, which promote cell proliferation and resistance to cell death [13].

Under hypoxia, cancer cells upregulate CAIX to regulate pH and survive acidic stress, resulting in high expression in many solid tumours but low levels in normal tissues. This dysregulation is associated with metabolic changes and apoptosis, which involves cytochrome c release and caspase-3 activation [14,15,16,17,18]. Therefore, targeting the Akt pathway, inhibiting CAIX-mediated pH regulation, and promoting cytochrome c release and caspase-3 activation are important strategies for developing new anticancer therapies [19,20].

MicroRNAs (miRNAs) are small non-coding RNAs that play an essential role in the post-transcriptional regulation of gene expression and are increasingly recognized as critical modulators of cancer development, progression, and therapeutic response [21]. Among the many dysregulated miRNAs in cancer, miR-21, miR-34a, and miR-210 are notable for their involvement in signalling pathways related to cell proliferation, apoptosis, and adaptation to cellular stress [22,23]. miR-21, a highly upregulated oncomiR, promotes tumour growth and drug resistance by suppressing genes such as phosphatase tensin homolog (*PTEN*) and programmed cell death 4 (*PDCD4*) [24]. In contrast, miR-34a functions as a tumour suppressor downstream of p53, contributing to apoptosis and cell cycle arrest, and is often reduced in malignant cells [25,26]. miR-210 is a central regulator of the hypoxia response, induced by HIF-1α and involved in mitochondrial function, angiogenesis, and survival under low-oxygen stress [27]. In anticancer drug development, especially when evaluating purine-based compounds, monitoring these miRNAs provides insight into mechanisms related to cell survival, apoptosis, and hypoxic adaptation. Their modulation in response to treatment may help clarify how purine derivatives exert their effects and support the development of more targeted cancer therapies.

**Figure 1:**
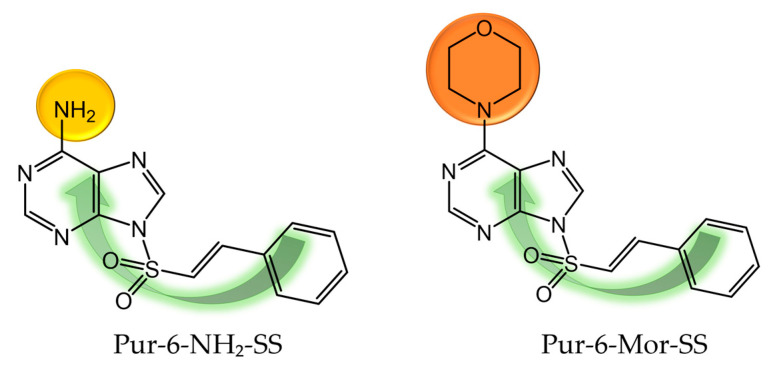
Molecular structure of Pur-6-NH_2_-SS ((*E*)-9-(styrylsulfonyl)-9*H*-purin-6-amine) and Pur-6-Mor-SS ((*E*)-6-morpholino-9-(styrylsulfonyl)-9*H*-purine) derivatives.

The aim of this study was to investigate the antitumor activity of Pur-6-NH_2_-SS ((*E*)-9-(styrylsulfonyl)-9*H*-purin-6-amine) and Pur-6-Mor-SS ((*E*)-6-morpholino-9-(styrylsulfonyl)-9*H*-purine) derivatives (Figure 1) [6,7] in 2D and 3D human cervical adenocarcinoma HeLa cell models to reveal how culture architecture influences drug response. Using a comprehensive multiparametric approach, we examined their impact on cell cycle progression, apoptosis, mitochondrial function, oxidative stress, and the regulation of key genes and microRNAs associated with cell survival, hypoxia, and apoptosis.

## 2. Results

### 2.1. Comprehensive Functional Analysis of HeLa Cells in 2D Culture Following Derivative Treatment

#### 2.1.1. Analysis of Cell Cycle Arrest in 2D HeLa Cells

**Figure 2:**
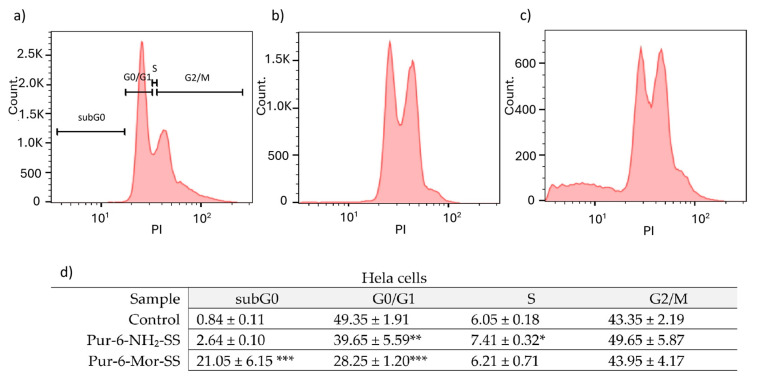
Flow cytometric analysis of the cell cycle distribution of HeLa cells exposed to 10 μM Pur-6-NH_2_-SS and Pur-6-Mor-SS. DNA histograms show changes in the cell cycle: (**a**) untreated control cells, (**b**) cells treated with Pur-6-NH_2_ -SS and (**c**) cells treated with Pur-6-Mor-SS. Control-derived gates were applied uniformly across all treatments. (**d**) Data are presented as percentage (%) of cells in the cell cycle phase. Statistically significant differences are labelled as follows: *** *p* < 0.0001; ** *p* = 0.003; * *p* = 0.002.

The effects of Pur-6-NH_2_-SS and Pur-6-Mor-SS derivatives on the cell cycle were examined in HeLa cells (Figure 2). In control cells, the highest percentage was observed in the G0/G1 phase (49.4 ± 1.9%), while 43.4 ± 2.2% of cells were in the G2/M phase. After treatment with the Pur-6-NH_2_-SS derivative, the proportion of cells in the G0/G1 phase decreased to 39.7 ± 5.6%, with 7.4 ± 0.3% in the S phase and 49.7 ± 5.9% in the G2/M phase. Treatment with the Pur-6-Mor-SS derivative increased the proportion of cells in the subG0 phase to 21.1 ± 6.2% and decreased the proportion in the G0/G1 phase to 28.3 ± 1.2%. The proportions of cells in the S and G2/M phases showed no changes compared to control cells.

#### 2.1.2. Assessment of Programmed Cell Death Induced by Derivatives in 2D HeLa Cultures

**Figure 3:**
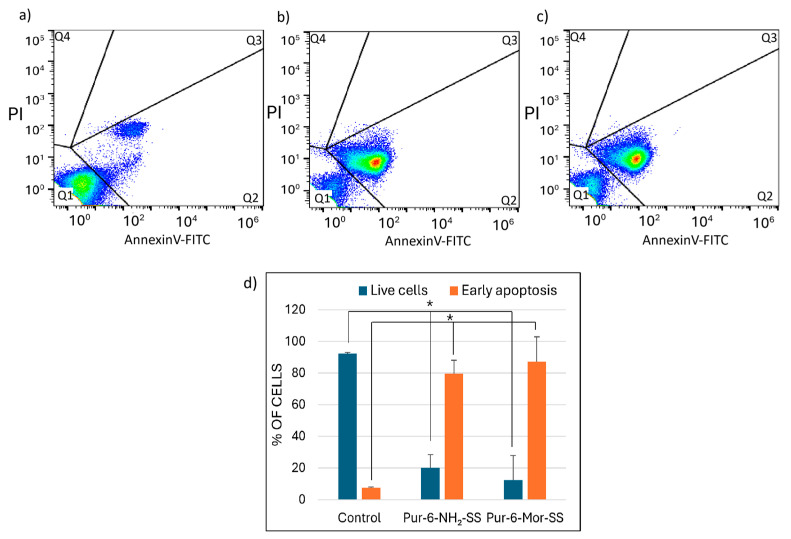
Detection of apoptosis in HeLa cells induced by 10 μM Pur-6-NH_2_-SS and Pur-6-Mor-SS after 24 h. The flow cytometric assay distinguishes viable cells (Q1), early apoptotic cells (Q2), and late apoptotic cells (Q3) and necrotic cells (Q4) by Annexin V/propidium iodide staining. (**a**) Dot plot of untreated control cells, (**b**) dot plot of cells treated with Pur-6-NH_2_-SS, (**c**) dot plot of cells treated with Pur-6-Mor-SS, (**d**) diagram of live and apoptotic cells. Statistically significant differences are labelled as * *p* < 0.0001.

The effects of Pur-6-NH_2_-SS and Pur-6-Mor-SS derivatives on apoptosis induction in HeLa cells were analysed by flow cytometry (Figure 3). After treatment with the Pur-6-NH_2_-SS derivative, 79.6 ± 8.5% of the cells underwent early apoptosis. Treatment with the Pur-6-Mor-SS derivative resulted in an even higher percentage of early apoptotic cells, at 87.4 ± 15.6%. The derivatives exerted their effects through a dominant accumulation of cells with a pronounced apoptotic green signal.

#### 2.1.3. Evaluation of Mitochondrial Dysfunction in HeLa Cells Following Treatment

**Figure 4:**
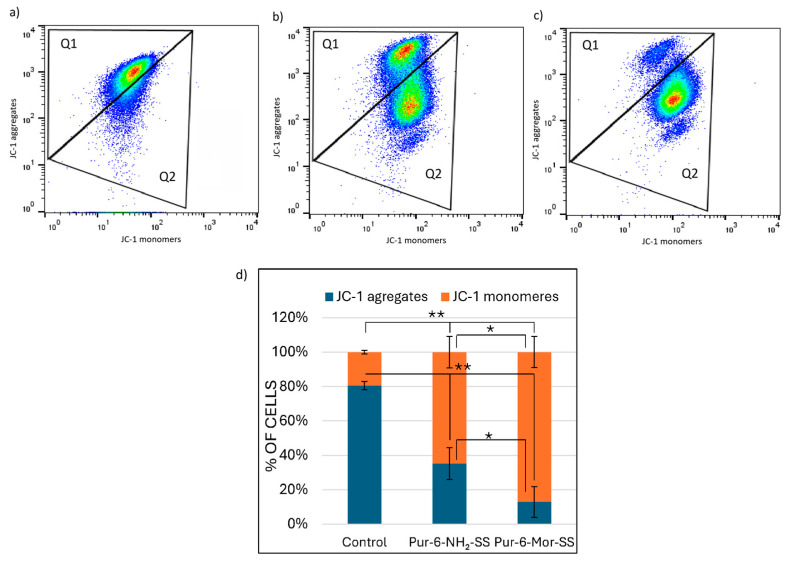
Detection of changes in mitochondrial membrane potential (ΔΨm) in HeLa cells after 24 h of treatment with 10 µM Pur-6-NH_2_-SS and Pur-6-Mor-SS derivatives. (**a**) Dot plot of control cells not treated, (**b**) dot plot of cells treated with Pur-6-NH_2_-SS, (**c**) dot plot of cells treated with Pur-6-Mor-SS, (**d**) diagram showing cells with JC-1 aggregates exhibiting red fluorescence, representing cells with intact mitochondria (Q1), and JC-1 monomers exhibiting green fluorescence, representing cells with disrupted mitochondria (Q2). Statistically significant differences are labelled ** *p* < 0.0001; * *p* = 0.002.

The change in mitochondrial membrane potential (∆Ψm) in HeLa cells after treatment with Pur-6-NH_2_-SS and Pur-6-Mor-SS derivatives was monitored by measuring JC-1 dye fluorescence as an indicator of mitochondrial integrity (Figure 4). Both derivatives caused a change in ∆Ψm in most cells. Treatment with the Pur-6-NH_2_-SS derivative resulted in a change in ∆Ψm in 64.3 ± 9.0% of cells, while mitochondrial potential was maintained in 35.0 ± 9.1% of cells. The effect of the Pur-6-Mor-SS derivative was more pronounced, with a change in ∆Ψm observed in 86.8 ± 9.0% of HeLa cells.

#### 2.1.4. Oxidative Stress Response in HeLa Cells Following Derivative Exposure

**Figure 5:**
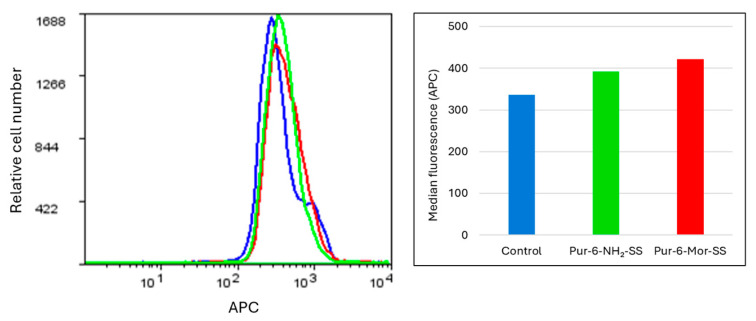
Determination of intracellular oxidative stress in HeLa cells exposed to Pur-6-NH_2_-SS and Pur-6-Mor-SS derivatives. The derivatives were applied at a concentration of 10 µM. The fluorescence histogram displays median values. Control (untreated) cells are shown in blue, cells treated with Pur-6-NH_2_-SS in green, and cells treated with Pur-6-Mor-SS in red.

Intracellular accumulation of reactive oxygen species (ROS), reflecting the level of cellular stress, was measured after one hour of exposing HeLa cells to Pur-6-NH_2_-SS and Pur-6-Mor-SS derivatives. Treated cells showed increased fluorescence compared to control (untreated) cells, indicating elevated intracellular ROS accumulation. As shown in Figure 5, the highest ROS level was observed in cells exposed to Pur-6-Mor-SS compared to those treated with Pur-6-NH_2_-SS, suggesting that Pur-6-Mor-SS induces a stronger oxidative stress response.

### 2.2. Integrative Analysis of CA IX Activity and Gene/miRNA Expression in 2D and 3D HeLa Cultures

#### 2.2.1. Quantification of Carbonic Anhydrase IX Activity in HeLa Cells Using ELISA

**Figure 6:**
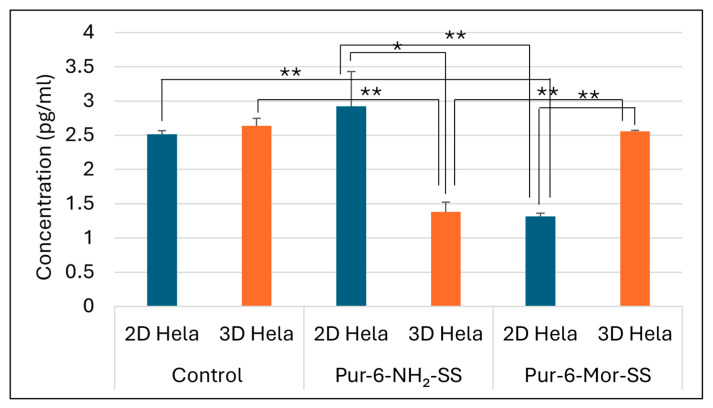
Detection of human CA IX concentration in 2D and 3D HeLa cells. The derivatives Pur-6-NH2-SS and Pur-6-Mor-SS were applied at a concentration of 10 μM for 24 h. Statistically significant differences are labelled ** *p* < 0.0001; * *p* = 0.002.

The concentration of carbonic anhydrase IX (CA IX) was determined by ELISA in the supernatant of HeLa cells grown in 2D and 3D cultures. The selected Pur-6-NH2-SS and Pur-6-Mor-SS derivatives showed different effects on CA IX concentration depending on the type of cell culture (Figure 6). In untreated control cells, no significant difference in CA IX concentration was observed between 2D and 3D cultures. Treatment with the Pur-6-NH2-SS derivative led to a slight increase in CA IX concentration in the 2D culture, with a measured value of 2.9 ± 0.5 pg/mL compared to the control. In contrast, a decrease in CA IX concentration of about 50%, to a value of 1.3 ± 0.1 pg/mL, was observed in the 2D culture treated with the Pur-6-Mor-SS derivative. In the 3D culture, the CA IX concentration after treatment with the Pur-6-NH2-SS derivative was 1.4 ± 0.1 pg/mL, which was lower than in the control. Treatment with the Pur-6-Mor-SS derivative did not change the CA IX concentration in spheroids compared to untreated cells.

#### 2.2.2. Expression Analysis of Apoptotic and Hypoxia-Related Genes in Treated HeLa Cells

**Figure 7:**
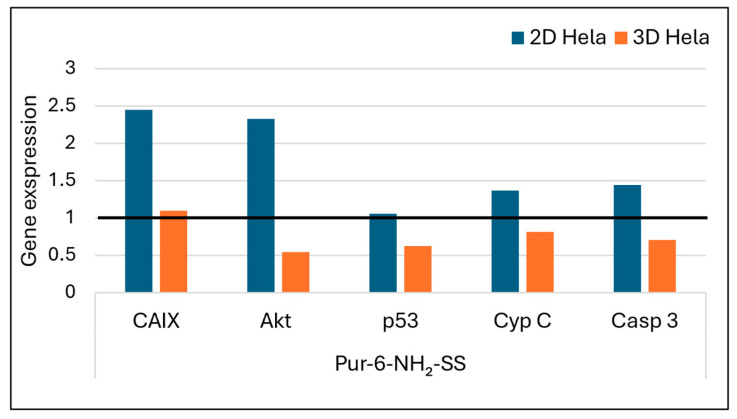
Gene expression of *Akt*, *CAIX*, *p53*, *caspase 3*, and *cytochrome C* after exposure to Pur-6-NH2-SS at a concentration of 5 µM in HeLa cells cultured in 2D and 3D for 24 h. Expression levels of the analysed genes were normalized to the reference gene *GAPDH*. Expression in control (untreated) cells is indicated by the black line.

**Figure 8:**
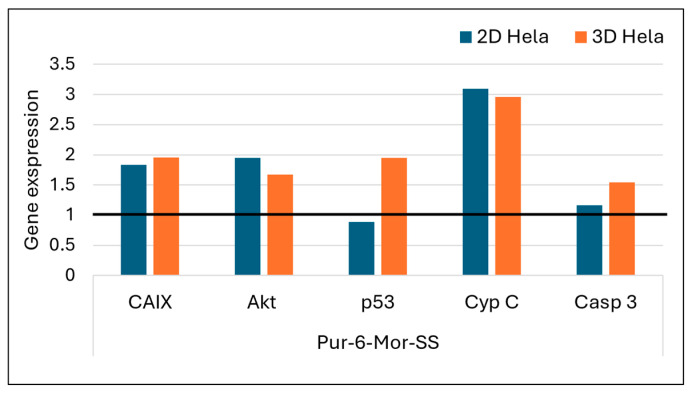
Gene expression of *Akt*, *CAIX*, *p53*, *caspase 3*, and *cytochrome C* after exposure to Pur-6-Mor-SS at a concentration of 5 µM in HeLa cells cultured in 2D and 3D for 24 h. Expression levels of the analysed genes were normalized to the reference gene *GAPDH*. Expression in control (untreated) cells is indicated by the black line.

The effects of Pur-6-NH2-SS and Pur-6-Mor-SS derivatives (Figure 7 and Figure 8) on the expression of *CAIX*, *Akt*, *p53*, *cytochrome C*, and *caspase 3* genes in HeLa cells grown in 2D and 3D cultures were analysed by RT-PCR. Increased expression of *Akt* and *CAIX* genes was detected in 2D HeLa cells treated with the Pur-6-NH2-SS derivative. In 3D HeLa cell cultures, *Akt* gene expression was reduced after the same treatment, while *CAIX* gene expression remained at the level of untreated control cells. The expression of the *p53* gene in the 2D culture did not change, but it was reduced in the 3D culture. *Caspase 3* and *cytochrome C* expression increased in the 2D culture, while a decrease in the expression of these genes was observed in the 3D culture.

Treatment with the Pur-6-Mor-SS derivative led to increased expression of all analysed genes in the 3D HeLa cell culture. In the 2D culture, *cytochrome C* expression is most pronounced, while the levels of *Akt* and *CAIX* genes are nearly the same. After treatment with the Pur-6-Mor-SS derivative in the 2D culture, a slight reduction in *p53* gene expression and a slight increase in *caspase 3* expression were also observed.

#### 2.2.3. Regulation of Key microRNAs Involved in Apoptosis and Hypoxia in HeLa Cells

**Figure 9:**
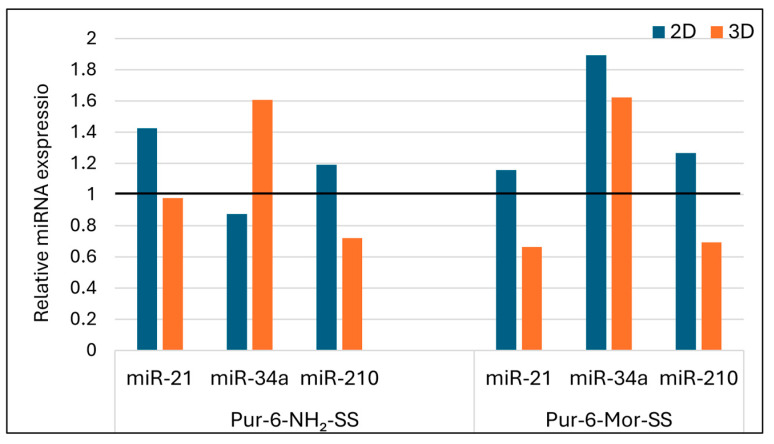
Relative expression of microRNAs in HeLa cells after treatment with Pur-6-NH_2_-SS and Pur-6-Mor-SS derivatives. The derivatives were applied at a concentration of 5 µM for 24 h. Expression levels were normalized to the reference miRNA miR-25. The black line represents control (untreated) cells.

Treatment with the Pur-6-NH_2_-SS derivative resulted in upregulated expression of miR-21 in HeLa cells grown in 2D culture, while no significant change was observed in 3D culture. For miR-34a, the Pur-6-NH_2_-SS derivative caused downregulation in 2D cultures and upregulation in 3D spheroids, indicating an inverse expression pattern depending on the structural organization of the cell model. miR-210 expression was elevated in 2D cells and reduced in 3D spheroids, suggesting a possible link between this miRNA and oxidative stress or hypoxic conditions (Figure 9).

Treatment with the Pur-6-Mor-SS derivative (Figure 9) led to a slight increase in miR-21 expression in 2D cultures, while a decrease was observed in 3D spheroids. For miR-34a, elevated expression was detected in both HeLa models, regardless of culture type. miR-210 expression was upregulated in 2D cultures and downregulated in 3D spheroids, further supporting the potential role of this derivative in modulating hypoxia-related responses and mitochondrial activity.

## 3. Discussion

Cancer remains a major global health burden, prompting the search for improved treatments. Purine analogues are promising candidates because their structural similarity to natural purines enables them to disrupt key cellular processes [28]. Our previous studies have shown that 6-morpholino and 6-amino-9-sulfonylpurine derivatives exhibit significant antitumour activity against human leukaemia and lymphoma cells [6,7]. In this study, our research has been extended to epithelioid tumour cells of the cervix: HeLa cells, a widely used model for studying tumour biology and drug response. The biological activity of the Pur-6-NH_2_-SS and Pur-6-Mor-SS derivatives was examined in both traditional 2D HeLa cell cultures and 3D spheroid models, which more closely mimic the tumour microenvironment. Using these complementary systems, the compounds were shown to disrupt the cell cycle, induce apoptosis, impair mitochondrial function, and alter the expression of key genes and miRNAs. The 3D spheroids also provided insight into drug penetration, resistance, and complex tumour-like cellular responses, resulting in a more comprehensive understanding of the derivatives’ antitumour potential. Treatment with the Pur-6-NH_2_-SS derivative resulted in a significant decrease in the proportion of cells in the G0/G1 phase, with a simultaneous increase in the number of cells in the S and G2/M phases, indicating inhibition of cell cycle progression and possible retention of cells in the phases of active replication and mitosis. Compared to the Pur-6-NH_2_-SS derivative, the Pur-6-Mor-SS derivative showed an even more pronounced cytotoxic effect on HeLa cells. Treatment with Pur-6-Mor-SS led to a significant accumulation of cells in the sub-G0 phase, indicating DNA fragmentation and advanced apoptosis [29,30]. At the same time, a decrease in the proportion of cells in the G0/G1 phase was observed, confirming a more pronounced cytostatic effect.

Mitochondrial dysfunction triggers the intrinsic apoptosis pathway by releasing proapoptotic factors and increasing ROS levels, which further damage cells and intensify apoptosis, especially in tumour cells [31,32,33]. Based on this, the prevalence of apoptotic cells was analysed in combination with measurements of changes in mitochondrial membrane potential and ROS accumulation. Apoptosis analysis showed activation of programmed cell death in about 80% of cells, confirming the strong proapoptotic effect of Pur-6-NH_2_-SS compounds. Disruption of mitochondrial membrane potential was observed in about 65% of treated cells, indicating involvement of the mitochondrial pathway of apoptosis [34,35,36]. Concurrently, accumulation of ROS was observed, further confirming activation of oxidative stress as a part of the mechanism of action. Apoptosis was more pronounced with Pur-6-Mor-SS than with Pur-6-NH_2_-SS derivatives, and mitochondrial potential was impaired in more than 85% of treated cells, further confirming strong activation of the mitochondrial pathway of apoptosis. Increased intracellular ROS signalling was also observed, indicating stronger oxidative stress after treatment with Pur-6-Mor-SS derivatives.

The results indicate that both types of purine-based derivatives exert cytotoxic effects on HeLa cells through multiple mechanisms, including cell cycle arrest, induction of apoptosis, mitochondrial dysfunction, and oxidative stress. These effects align with findings for similar purine analogues, such as roscovitine, a CDK-inhibiting purine derivative, which induced apoptosis in HeLa cells by decreasing mitochondrial membrane potential, modulating Bcl-2 family proteins, and inducing autophagy following prooxidant stress [30].

Further confirmation of apoptosis promotion was obtained by measuring the gene expression of *cytochrome C* and *caspase 3*, the main executioner caspase in the cells [37]. Treatment of HeLa cells in 2D culture with the purine-based derivatives led to increased gene expression of *cytochrome C* and *caspase 3*, indicating activation of the mitochondrial-mediated (intrinsic) apoptosis pathway [37]. In both cases, this process was confirmed by disruption of the mitochondrial membrane potential, suggesting that the compounds destabilise mitochondrial function and cause the release of cytochrome C and activation of the caspase cascade.

Different response patterns were observed in 3D HeLa spheroids. The derivative Pur-6-NH_2_-SS resulted in reduced expression of *cytochrome C* and *caspase 3*, indicating resistance of the three-dimensional cell architecture to apoptosis induction. This resistance may be due to limited drug penetration, hypoxic conditions within the spheroids, or activation of protective signalling pathways, which together help maintain mitochondrial stability and reduce the apoptotic response [38,39,40]. In contrast, the Pur-6-Mor-SS derivative showed increased expression of the same proapoptotic genes in the 3D model, suggesting that this agent also effectively induces mitochondrial stress and apoptosis in a more physiologically relevant three-dimensional tumour environment, which is typically more resistant to therapy [41,42].

CAIX is a hypoxia-induced enzyme that supports tumour pH regulation and progression [43,44,45], and its inhibition can suppress tumour growth [16,46,47], by using small-molecule sulfonamide or sulfamate inhibitors that block its zinc-dependent activity [48]. In this study, the effects of the Pur-6-NH_2_-SS derivative differed between 2D and 3D models of HeLa cells. In 2D culture, CAIX concentration was almost twice as high as in 3D spheroids, with simultaneously increased expression of *CAIX*, the *Akt* gene, and miR-210—known targets of the HIF-1α signalling pathway [49]. These results suggest that this derivative does not inhibit CAIX via the Akt/HIF signalling pathway; rather, its effect may be linked to alternative mechanisms or compensatory cellular responses to stress. In contrast, the same compound in 3D HeLa spheroids led to decreased *Akt* gene expression, unchanged *CAIX* expression, and lower miR-210 levels, which in turn resulted in lower CAIX levels. These findings suggest reduced activation of the hypoxia-related signalling pathway and potential inhibition of proliferation signalling, despite the inherent resistance of spheroids to therapy. The reduced CAIX activation in spheroids could be due to limited drug diffusion, hypoxic gradients within the spheroids, or altered cell dynamics leading to suppression of Akt signalling involved in HIF-1α stability and transcription [50,51].

The expression of miR-21 is positively related to the activation of the PI3K/Akt signalling pathway, as miR-21 inhibits the tumour suppressor gene PTEN, thereby enabling phosphorylation and activation of the Akt protein [52]. Indirectly, this connection could influence the expression of CAIX, as the *CAIX* gene is under the transcriptional control of the Akt/HIF-1α axis, which is frequently activated in tumour cells. Based on this correlation, the gene expression of miR-21 was analysed in HeLa cells after treatment with purine-based derivatives. The results showed increased expression of miR-21 in the 2D culture of HeLa cells after treatment with both purine-based derivatives, while in 3D spheroids there was a decrease in expression, with the effect of the Pur-6-Mor-SS derivative being more pronounced. MiR-21 is an oncomiR whose overexpression promotes cancer progression by suppressing tumour-suppressor genes such as PTEN, PDCD4, and RECK, leading to increased proliferation, invasiveness, and resistance to apoptosis [53]. In 2D cultures, increased miR-21 expression likely reflects a stress-induced survival response to purine-based derivatives, whereas in 3D spheroids miR-21 was suppressed—especially by Pur-6-Mor-SS—possibly due to stronger proapoptotic activity or reduced PI3K/Akt signalling, which normally regulates miR-21 [54,55]. The reduced expression of miR-21 in spheroids may reflect increased sensitivity of tumour cells to the therapeutic effect of the derivatives and a reduced ability to avoid apoptosis, further highlighting the importance of using 3D models in the preclinical evaluation of antitumour drugs.

In HeLa cells, the HPV-18 E6 oncoprotein binds to p53 and promotes its degradation, thereby suppressing normal tumour-suppressor function [56,57]. In this study, reduced expression of p53 was observed in HeLa spheroids after treatment with the derivative Pur-6-NH_2_-SS, while *p53* expression in 2D culture did not change significantly. A similar pattern was observed with Pur-6-Mor-SS in 2D culture, where *p53* expression was slightly lower. However, a notable increase in *p53* expression was observed in 3D spheroids treated with the Pur-6-Mor-SS derivative, which may indicate reactivation of p53 transcription under cellular stress in a three-dimensional model. The relationship between p53 and miR-34a, one of the best-known miRNAs of the tumour suppressor family miR-34, is also important. miR-34a is a direct transcriptional target of p53, and its expression increases in response to DNA damage, apoptosis activation, and cell cycle arrest in G1 phase [58,59,60]. Accordingly, in this work, an increase in miR-34a expression was observed in 2D and 3D HeLa cells after treatment with the Pur-6-Mor-SS derivative, correlating with the previously reported increase in *p53* in spheroids. Interestingly, Pur-6-NH_2_-SS induced an increase in miR-34a exclusively in 3D spheroids, in parallel with an increase in *p53*. This further supports activation of the p53/miR-34 axis under conditions where the tumour model reflects physiological hypoxia and resistance in a more complex manner.

MiR-210 regulates processes including proliferation, migration, apoptosis, differentiation, DNA repair, and metabolism, and is best known as a key mediator of the hypoxic response [61,62]. The tested derivatives produced the same effect on 2D and 3D HeLa cells, with increased expression of miR-210 in the 2D model and reduced expression in the 3D model. Although elevated miR-210 expression in cell spheroids was considered a possible mechanism for greater resistance to the tested derivatives, the results suggest that resistance in cell spheroids is promoted by other proliferative stimulating factors.

Overall, the results indicate that both purine-based derivatives exert cytotoxic effects on HeLa cells through mechanisms such as cell cycle arrest, induction of apoptosis, impairment of mitochondrial function, and oxidative stress, with a stronger effect observed for the Pur-6-Mor-SS derivative. Although HeLa cell spheroids exhibited greater resistance to the tested derivatives, the effects of Pur-6-NH_2_-SS were still evident in altered Akt/HIF-related signalling markers, suggesting a possible impact on this pathway rather than definitive inhibition. In contrast, Pur-6-Mor-SS had a different effect, showing a more pronounced impact on the 2D model and weaker inhibition in the 3D culture.

## 4. Materials and Methods

### 4.1. Cell Cultivation and Treatment

HeLa cells were maintained at 37 °C in a humidified atmosphere containing 5% CO_2_ using a CO_2_ incubator (IGO 150 CELLlife™, JOUAN, Thermo Fisher Scientific, Waltham, MA, USA). Cells were grown in Dulbecco’s Modified Eagle Medium (DMEM; Lonza, Basel, Switzerland) complemented with 10% heat-inactivated fetal bovine serum (FBS; GIBCO Invitrogen, Paisley, UK), 2 mM L-glutamine, and a standard antibiotic mixture (100 U/mL penicillin and 100 µg/mL streptomycin; GIBCO Invitrogen, Paisley, UK). When cultures reached confluence, they were detached using a 0.25% trypsin–EDTA solution (GIBCO Invitrogen, Paisley, UK) and then processed for the respective experimental procedures.

HeLa cells were treated with the Pur-6-NH_2_-SS and Pur-6-Mor-SS derivatives, which were synthesised and characterised at the Laboratory for Biomolecular Interactions and Spectroscopy, Division of Organic Chemistry and Biochemistry, Ruđer Bošković Institute.

### 4.2. Cell Cycle Distribution

HeLa cells were seeded in 6-well plates at a density of 3 × 10^5^ cells per well. Cells were treated with Pur-6-NH_2_-SS and Pur-6-Mor-SS derivatives at a final concentration of 10 μM for 24 h. After incubation, the cells were collected and fixed with 70% cold ethanol. The fixed cells were stored at −20 °C until analysis. On the day of analysis, the cells were centrifuged and washed with PBS. Prior to staining, the cells were incubated with RNase A at a final concentration of 0.2 µg/µL for 5 min at room temperature. The cells were stained with propidium iodide (PI) at a final concentration of 15 µg/mL for 30 min at room temperature. After staining, the cells were transferred to flow cytometry tubes and analysed using a FACS Canto II flow cytometer (BD Biosciences, Franklin Lakes, NJ, USA) with FlowJo software version 10.10.0 (FlowJo LLC, Ashland, OR, USA).

### 4.3. Apoptosis Analysis

HeLa cells were seeded in 6-well plates at a density of 3 × 10^5^ cells per well and incubated overnight in a CO_2_ incubator. The next day, the cells were treated with Pur-6-NH_2_-SS and Pur-6-Mor-SS derivatives at a final concentration of 10 µM for 24 h. In parallel, cells were incubated with doxorubicin as a positive control at a final concentration of 3 μM. Cells were collected, washed with PBS, and the pellet was resuspended in 500 µL of 1× binding buffer from the Annexin V-FITC Apoptosis Detection Kit (Abcam, Cambridge, UK, EU). The stained cells were analysed using a FACS Canto II flow cytometer (BD Biosciences, Franklin Lakes, NJ, USA), and the data were processed using FlowJo software version 10.10.0 (FlowJo LLC, Ashland, OR, USA).

### 4.4. Measurement of Changes in the Mitochondrial Membrane

HeLa cells were seeded in 6-well plates at a density of 3 × 10^5^ cells per well. The following day, cells were treated with Pur-6-NH_2_-SS and Pur-6-Mor-SS derivatives at a final concentration of 10 µM for 24 h. After incubation, cells were collected, centrifuged at 600× *g* for 4 min at 4 °C, and resuspended in complete medium containing JC-1 dye prepared according to the manufacturer’s instructions (Mitochondria Staining Kit for Mitochondrial Potential Changes Detection, Sigma-Aldrich, St. Louis, USA). Cells were incubated with JC-1 dye for 20 min in a CO_2_ incubator. After incubation, the JC-1 solution was removed, and the cells were resuspended in JC-1 buffer. Analysis was performed using a FACS Canto II flow cytometer (BD Biosciences, Franklin Lakes, NJ, USA), and data were processed using FlowJo software version 10.10.0 (FlowJo LLC, Ashland, OR, USA).

### 4.5. Reactive Oxygen Species (ROS) Detection

HeLa cells were resuspended in PBS at a concentration of 5 × 10^5^ cells/mL and incubated for 1 h at 37 °C with 5% CO_2_ in the presence of Pur-6-NH_2_-SS and Pur-6-Mor-SS derivatives at a final concentration of 10 µM. After incubation, the Red ROS fluorescent probe from ROS detection reagent was added to the cells according to the manufacturer’s instructions (Fluorometric Intracellular ROS Kit, Sigma-Aldrich, St. Louis, USA), and the cells were incubated for a further 30 min under the same conditions (37 °C, 5% CO_2_). Analysis was performed using a FACS Canto II flow cytometer (BD Biosciences, Franklin Lakes, NJ, USA), and the data were processed using FlowJo software version 10.10.0 (FlowJo LLC, Ashland, OR, USA).

### 4.6. Formation of 3D Cells

HeLa cells cultured in a monolayer were detached with trypsin, resuspended in complete medium, and seeded into 96-well V-bottom plates (Brand, Wertheim, Germany). Spheroid conditions were optimised by testing different seeding densities and culture durations, with microscopy used to identify the parameters that consistently produced a compact 400 µm spheroid for further experiments. Based on this optimization, cells were seeded at 1 × 10^4^ cells/mL, centrifuged for 10 min at 1000 rpm at room temperature, and allowed to form spheroids in a CO_2_ incubator at 37 °C for 72 h. After incubation, the spheroids were treated with Pur-6-NH_2_-SS and Pur-6-Mor-SS derivatives. The treated spheroids were then used to investigate antiproliferative activity by MTT assay, carbonic anhydrase IX (CA IX) activity, and miRNA and gene expression analysis by RT-PCR.

### 4.7. Determination of CAIX Concentration in Supernatant

The concentration of human carbonic anhydrase IX (CAIX) was measured using an ELISA-based immunoenzymatic assay (Human Carbonic Anhydrase IX ELISA Kit, Sigma-Aldrich, St. Louis, MO, USA) based on specific antigen–antibody interactions in cell culture supernatant. HeLa cells cultured under 2D conditions were seeded in 96-well plates at 1 × 10^5^ cells/mL, while cells grown in 3D culture were seeded at 1 × 10^4^ cells/mL, following the previously described protocol. Cells were treated for 24 h with Pur-6-NH_2_-SS and Pur-6-Mor-SS derivatives at a final concentration of 10 µM. After incubation, culture supernatants were collected and used to determine CAIX concentration according to the manufacturer’s instructions.

### 4.8. RNA Isolation

HeLa cells cultured in 2D were seeded in cell culture flasks at a concentration of 1 × 10^6^ cells, while 3D HeLa cultures were seeded in 96-well plates at a concentration of 1 × 10^4^ cells/mL as described in the protocol. Cells were treated with Pur-6-NH_2_-SS and Pur-6-Mor-SS derivatives at a final concentration of 5 µM for 24 h. After incubation, the 2D cells and 3D spheroids (96 spheroids) were collected and lysed, and total RNA was isolated using the RNeasy Mini Kit (Qiagen, Hilden, Germany). The isolated RNA was then used for RT-PCR quantification and microRNA expression analysis.

### 4.9. Gene Expression

cDNA was synthesised from total RNA using the PrimeScript 1st Strand cDNA Synthesis Kit (Takara Bio EU, Saint-Germain-en-Laye, France) according to the manufacturer’s instructions. Subsequent amplification was performed on a QuantStudio 5 Real-Time PCR system (Thermo Fisher Scientific, Rockford, IL, USA). PCR reactions (20 μL total volume) were prepared according to the GoTaq Flexi DNA Polymerase protocol (Promega, Madison, WI, USA). The thermal profile included an initial denaturation at 95 °C for 2.5 min, followed by 40 cycles at 95 °C for 30 s and gene-specific annealing for 1.5 min (p53 at 61 °C; caspase-3, cytochrome c, Akt, and CA IX at 57 °C; GAPDH at 68 °C). Gene-specific primers were used for p53 (F: 5′-GAT GCT GTC CGC GGA CGA TAT-3′; R: 5′-CGT GCA AGT CAC AGA CTT GGC-3′), caspase-3 (F: 5′-TCG GTC TGG TAC AGA TGT CG-3′; R: 5′-CAT ACA AGA AGT CGG CCT CC-3′), cytochrome c (F: 5′-AGA ACA AAG GCA TCA TCT GGG-3′; R: 5′-TCA GCT GTA GCC GAG AGT CA-3′), Akt (F: 5′-CTT CCT CAC AGC CCT GAA GT-3′; R: 5′-TAA TGT GCC CGT CCT TGT CC-3′), and CA IX (F: 5′-TGA GGA AGG CTC AGA GAC TCA-3′; R: 5′-TCA GCT GTA GCC GAG AGT CA-3′), with GAPDH (F: 5′-CCA TCA ATG ACC CCT TCA TTG ACC-3′; R: 5′-GAA GGC CAT GCC AGT GAG CTT CC-3′) as the housekeeping (referent) gene. The Ct values of the samples were normalized to GAPDH. Relative expression was calculated using the 2^−ΔΔCt^ method [63].

### 4.10. Detection of miRNAs

Total RNA was reverse transcribed according to the protocol supplied with the TaqMan Advanced miRNA cDNA Synthesis Kit (Thermo Fisher Scientific, Rockford, IL, USA). For the analysis, hsa-miR-25-3p and hsa-miR-93-5p [61] were used as reference miRNAs, while hsa-miR-21-5p, hsa-miR-34a-5p, and hsa-miR-210-3p were selected as target miRNAs. Quantification was performed using TaqMan miRNA assays on a QuantStudio 5 Real-Time PCR System (Thermo Fisher Scientific, Rockford, IL, USA). Relative changes in miRNA expression were calculated using the 2^−ΔΔCt^ method as described by Livak et al. [63], with normalisation to the reference miRNAs.

### 4.11. Statistical Analysis

All experiments were performed in duplicate or triplicate, with untreated cells as controls. Statistical comparisons between treatment groups and the control were conducted, where applicable, using one-way ANOVA with Bonferroni post hoc adjustments (*p* < 0.05). Data processing and statistical analyses were performed in XLSTAT version 2025.1.0 for Microsoft Excel.

## Data Availability

Data are available from the authors upon reasonable request.
